# Aberrant enteric neuromuscular system and dysbiosis in amyotrophic lateral sclerosis

**DOI:** 10.1080/19490976.2021.1996848

**Published:** 2021-11-23

**Authors:** Yongguo Zhang, Destiny Ogbu, Shari Garrett, Yinglin Xia, Jun Sun

**Affiliations:** a Department of Microbiology/Immunology, University of Illinois at Chicago, Chicago, USA; b Department of Medicine, Jesse Brown Va Medical Center, Chicago, USA

**Keywords:** Autoimmune disease, enteric neuromuscular system, FALS, *Lachnospiraceae bacterium A4*, butyrate-producing bacteria, neuromuscular disease, colonoids, protein aggregation, SALS

## Abstract

Amyotrophic Lateral Sclerosis is a neuromuscular disease characterized by the progressive death of motor neurons and muscle atrophy. The gastrointestinal symptoms in ALS patients were largely ignored or underestimated. The relationship between the enteric neuromuscular system and microbiome in ALS progression is unknown. We performed longitudinal studies on the enteric neuron system (ENS) and microbiome in the ALS human-SOD1^G93A^ (Superoxide Dismutase 1) transgenic mice. We treated age-matched wild-type and ALS mice with butyrate or antibiotics to investigate the microbiome and neuromuscular functions. We examined intestinal mobility, microbiome, an ENS marker GFAP (Glial Fibrillary Acidic Protein), a smooth muscle marker (SMMHC, Smooth Muscle Myosin Heavy Chain), and human colonoids. The distribution of human-G93A-SOD1 protein was tested as an indicator of ALS progression. At 2-month-old before ALS onset, SOD1^G93A^ mice had significantly lower intestinal mobility, decreased grip strength, and reduced time in the rotarod. We observed increased GFAP and decreased SMMHC expression. These changes correlated with consistent increased aggregation of mutated SOD1^G93A^ in the colon, small intestine, and spinal cord. Butyrate or antibiotics treated SOD1^G93A^ mice had a significantly longer latency to fall in the rotarod test, reduced SOD1^G93A^ aggregation, and enhanced enteric neuromuscular function. Feces from 2-month-old SOD1^G93A^ mice significantly enhanced SOD1^G93A^ aggregation in human colonoids transfected with a SOD1^G93A^-GFP plasmid. Longitudinal studies of microbiome data further showed the altered bacterial community related to autoimmunity *(e.g., Clostridium sp. ASF502, Lachnospiraceae bacterium A4)*, inflammation (e.g., *Enterohabdus Muris*,), and metabolism (e.g., *Desulfovibrio fairfieldensis*) at 1- and 2-month-old SOD1^G93A^ mice, suggesting the early microbial contribution to the pathological changes. We have demonstrated a novel link between the microbiome, hSOD1^G93A^ aggregation, and intestinal mobility. Dysbiosis occurred at the early stage of the ALS mice before observed mutated-SOD1 aggregation and dysfunction of ENS. Manipulating the microbiome improves the muscle performance of SOD1^G93A^ mice. We provide insights into the fundamentals of intestinal neuromuscular function and microbiome in ALS.

## Introduction

ALS is characterized by the progressive death of motor neurons and muscle atrophy. Early diagnosis of ALS has been a long-standing challenge in the field. Case reports indicate that celiac disease with neurologic manifestations has been misdiagnosed as ALS.^1–3^ Researchers in Israel discovered a possible link between ALS and sensitivity to gluten.^4^ Others have also reported that ALS syndrome may be associated with autoimmunity and gluten sensitivity. These reports suggest that ALS patients show gastrointestinal symptoms. Although the data are preliminary, gluten sensitivity is potentially treatable; the diagnostic challenge should not be overlooked. Moreover, inflammatory cytokines (e.g., IL-6) and bacterial lipopolysaccharides (LPS) were elevated in ALS.^5,6^ Autoimmune disease (e.g., Crohn’s disease) associations with ALS raise the possibility of shared genetic or environmental risk factors in the pathogenesis and progression of ALS.^7^

Altered intestinal homeostasis and microbiome contribute to various neurological diseases (e.g. autism, Alzheimer’s disease, and Parkinson’s disease). We are the first to report the elevated intestinal inflammation, reduced beneficial bacteria, and shift of microbiome profile in ALS.^8–10^ Later, there are studies from several groups reporting the dysbiosis in human ALS and experimental animal models.^11–16^ For example, Niccolai et al.^16^ compared the inflammatory and microbiota profile of ALS patients with healthy family caregivers in different clinical characteristics. They reported a distinct cytokine profile both at the systemic and intestinal level in patients as well as different clinical phenotypes and progression rates. The 16SrRNA metagenome analysis revealed slight differences in ALS patients, compared to controls as well as in patients with slow progression, marked by the reduced butyrate-producing bacteria . Thus, the current evidence indicates that intestinal dysfunction and dysbiosis may actively contribute to ALS pathogenesis.

Intestinal mobility is a key physiologic parameter governing digestion and absorption of nutrients affected by the ENS, microbiome, and host genetics.^17–21^ The ENS is an important regulator of the proliferation and differentiation of the mucosal epithelium. Serotonergic neurons in the myenteric plexus activate submucosal cholinergic neurons that innervate intestinal crypts to stimulate proliferation, thus playing an essential role in various neurodegenerative diseases.^22–24^ However, studies of the ENS, mobility, and microbiome in ALS are lacking.

In the current study, we examined the changes in enteric neurons and smooth muscle in the prodromal phase of ALS, using an ALS human-SOD^G93A^ transgenic G93A mice, with or without butyrate treatment. We chose human-SOD^G93A^ transgenic mice because a fraction of familial ALS is associated with mutations in the superoxide dismutase gene (SOD1).^25^ Mouse models expressing ALS-linked human-SOD1 mutations effectively recapitulate many features of the human disease and have been extensively used to investigate pathogenic mechanisms of ALS.^26^ We performed longitudinal studies on the ENS, microbiome, and SOD1 aggregation in the ALS G93A mice. Human colonoids transfected with SOD1^G93A^-GFP (Green Fluorescent Protein) plasmids were used for the microbial-host interactions and SOD1 aggregation. We investigated the mechanism in altered ENS and intestinal mobility that contribute to ALS progression, correlated with SOD1^G93A^ aggregation, and dysbiosis. Our data suggest that modulating the microbiome by treating the mice with beneficial butyrate or antibiotics changes the ENS and disease progression in ALS. This study provides insights into the fundamentals of intestinal neuromuscular structure/function and microbiome in ALS. A better understanding of the intestinal dysfunction and dysbiosis in ALS will help its early diagnosis and development of new treatments.

## Materials and methods

### Animals

SOD1^G93A27^ and age-matched wild-type mice were used in this study, SOD^G93A^ mice were originally purchased from Jackson Laboratory (B6SJL-Tg (SOD1-G93A) 1 Gur/J, stock No.002726). All experiments were carried out in strict accordance with the recommendation in the Guide for the Care and Use of Laboratory Animals of the National Institutes of Health. Mice were provided with water ad libitum and maintained on a 12-hour dark/light cycle. The protocol was approved by the IACUC of the University of Illinois at Chicago Committee on Animal Resources (ACC 18–233).

### Measurement of gastrointestinal transit times

SOD1^G93A^ and age-matched WT mice were used with six mice for each group at each time point. On the test day, mice were transferred to individual empty plastic cages (devoid of bedding) and were deprived of food, with free access to water. Two hours after food deprivation, mice were then gavaged with 150 μl of Evans blue marker (5% Evans blue, 5% gum Arabic in drinking water) between 09:00 and 09:30 local time. After gavage, mice were fed ad libitum. The mice were observed at 5 min intervals until feces with blue were eliminated (maximum time of observation was 450 min). The time from the end of gavaging to the first blue fecal pellet was measured in minutes and constituted the whole gut transit time.

### Rotarod test

SOD1^G93A^ and age-matched WT mice were used with six mice for each group at each time point. Motor coordination, strength, and balance were assessed using a rotarod (Harvard Apparatus, Holliston, MA). Mice started training on the rotarod daily for three days before recording the data. Animals were placed onto the rod at a speed of 4 rpm which accelerated over the course of 300 seconds to 40 rpm. Latency to fall was recorded when the mouse fell from the rod. Each mouse was tested in 4 trials per day for 2 consecutive days. The mean times for 8 trials of the tests were calculated for each mouse.

### Assessment of grip strength

SOD1^G93A^ and age-matched WT mice were used with six mice for each group at each time point. Forelimb and hindlimb grip measurements were acquired in triplicate with a 25 N Grip strength meter (Harvard Apparatus, Holliston, MA, USA). The mice were lowered onto a triangle bar of the grip strength meter until the animals gripped the bar with their forelimbs or hindlimbs, then the mice were pulled gently backward until they released their grip. The force gauge of the grip meter recorded the maximum force.

### Western blot analysis and antibodies

Mouse intestinal mucosal cells were collected by scraping from mouse colon, including proximal and distal colon, as previously described.^28^ Briefly, mouse mucosal cells were lysed in lysis buffer (1% Triton X-100 (Sigma-Aldrich, X100), 150 mM NaCl (J.T. Baker 3624–19), 10 mM Tris (Fisher Scientific, BP152-5) pH 7.4, 1 mM EDTA (Fisher Scientific, BP120-1), 1 mM EGTA (Sigma-Aldrich, E3889) pH 8.0, 0.2 mM sodium ortho-vanadate (Sigma-Aldrich, S6508) and protease inhibitor cocktail (Roche Diagnostics, 118,367,001). Cultured cells were rinsed twice in ice-cold Hanks’ balanced salt solution (Sigma-Aldrich, H1387), lysed in protein loading buffer (50 mM Tris, pH 6.8, 100 mM dithiothreitol (Amresco, 0281), 2% SDS (Sigma-Aldrich, L3771), 0.1% bromophenol blue (IBI Scientific, IB74040) and 10% glycerol (Sigma-Aldrich, G5516)) and sonicated (Branson Sonifier, 250). Equal amount of protein was separated by SDS-polyacrylamide gel electrophoresis, transferred to nitrocellulose (Bio-rad, 162–0112) and immunoblotted with primary antibodies: anti-GFAP (Abcam, ab53554), SMMHC (Abcam, ab53219), human SOD-1^58^ or β-actin (Sigma-Aldrich, A1978) antibodies and visualized by ECL chemiluminescence (Thermo Scientific, 32,106). Membranes probed with more than one antibody were stripped before re-probing. Western blot bands were quantified using Image Lab 4.01 (Bio-Rad).

### Immunofluorescence

Intestinal and Lumbar spine tissues were freshly isolated and paraffin-embedded after fixation with 10% neutral-buffered formalin. Immunofluorescence was performed on paraffin-embedded sections (5 μm). After preparation of the slides as described previously,^28^ tissue samples were incubated with anti-GFAP (Abcam, ab53554), SMMHC (Abcam, ab53219), α-SMA (Abcam, ab5694), PGP9.5 (Invitrogen, MA1-90,008), human SOD-1 at 4°C overnight. Samples were then incubated with sheep anti-goat Alexa Fluor 594 (Life Technologies, A11058), or goat anti-mouse Alexa Flour 488 (Life Technologies, A-11001) and DAPI (Life Technologies, D1306) for 1 h at room temperature. Tissues were mounted with SlowFade (Life Technologies, s2828), followed by a coverslip, and the edges were sealed to prevent drying. Specimens were examined with Zeiss laser scanning microscope (LSM) 710. Fluorescence intensity was determined by using Image J software. This method determines the corrected total fluorescence by subtracting out background signal, which is useful for comparing the fluorescence intensity between cells or regions.

### Immunohistochemistry

After preparation of the slides, antigen retrieval was achieved by incubation of the slides for 15 min in the hot preheating sodium citrate (pH 6.0) buffer, and 30 min of cooling at room temperature. Endogenous peroxidases were quenched by incubating the slides in 3% hydrogen peroxide for 10 min, followed by three rinses with HBSS, and incubation for 1 hour in 3% BSA + 1% goat serum in HBSS to reduce nonspecific background. Primary antibodies anti-GFAP (Abcam, ab53554) or human SOD-1 were applied overnight in a cold room. After three rinses of the slides with HBSS, they were incubated in secondary antibodies (1:100, Jackson ImmunoResearch Laboratories, 115–065-174) for 1 hour at room temperature. After washing with HBSS for 10 minutes, the slides were incubated with vectastain ABC reagent (Vector Laboratories, PK-6100) for 1 hour. After washing with HBSS for five minutes, color development was achieved by applying a peroxidase substrate kit (Vector Laboratories, SK-4800) for 2 to 5 minutes, depending on the primary antibody. The duration of peroxidase substrate incubation was determined through pilot experiments and was then held consistently for all slides. After washing in distilled water, the sections were counterstained with hematoxylin (Leica, 3,801,570), dehydrated through ethanol and xylene, and cover slipped using a permount (Fisher Scientific, SP15-100). Immunohistochemistry intensity was also determined by using Image J software.

### Butyrate treatment in mice

SOD1^G93A^ mice were divided into two groups randomly, six mice for non-treatment group and 10 mice for butyrate treatment group. The butyrate-treated group receives sodium butyrate (Sigma-Aldrich, 303,410) at a 2% concentration in filtered drinking water. Control group receives filtered drinking water without sodium butyrate. All animals are weighted and received detailed clinical examination, which included appearance, movement and behavior patterns, skin and hair conditions, eyes and mucous membranes, respiration, and excreta. If restricted outstretching of the hind legs is observed when the tail is held, it means the symptom of ALS is obvious. Lie the mouse on the back, if it cannot turn over in 30 seconds, the mouse is humanely sacrificed.

### Antibiotic treatment in mice

SOD1^G93A^ mice were divided into two groups randomly, eight mice for non-treatment group, and 11 mice for antibiotic treatment group. The antibiotic-treated group receives antibiotics (1 mg/ml metronidazole and 0.3 mg/ml clindamycin) in filtered drinking water. Control group receives filtered drinking water without antibiotics. All animals are weighted and received detailed clinical examination, which included appearance, movement and behavior patterns, skin and hair conditions, eyes and mucous membranes, respiration and excreta. If restricted outstretching of the hind legs is observed when the tail is held, it means the symptom of ALS is obvious. Lie the mouse on the back, if it cannot turn over in 30 seconds, the mouse is humanely sacrificed.

### Human colonoid transfection and treatment with SOD1^G93A^ mice feces

Human organoids were developed using endoscopy samples in the UIC hospital, as we did in previous studies.^29,30^ Crypts were released from colon tissue by incubation for 30 min at 4°C in phosphate-buffered saline containing 2 mmol/L EDTA. Isolated crypts were counted and pelleted. A total of 500 crypts were mixed with 50 μL Matrigel (BD Biosciences, San Jose, CA) and plated in 24-well plates. The colonoids were maintained in Human IntestiCult Organoid Growth Medium (STEMCELL Technologies, Inc, Vancouver, BC). The human colonoids were transfected with the plasmids for fluorescent protein tagged human ALS-causing mutation SOD1^G93A^-GFP. Forty-eight hours after transfection, the human organoids were treated with SOD1^G93A^ mice feces.

Fresh feces were collected from 5 SOD1^G93A^ mice and then well mixed. A total of 100 mg feces were homogenized in 6 mL Hanks Balanced Salt Solution and centrifuged for 30 seconds at 300 rpm, at 4°C, to pellet the particulate matter. Organoids were treated with 250 μL feces supernatant for 2 hours, the organoids were washed 3 times with Hanks Balanced Salt Solution, and then the cells were incubated in regular organoid culture medium for 2 hours. The percentage of cells with human-SOD1 protein aggregates was counted in each experimental group.

### Microbiome 16S rRNA sequencing and bioinformatic analysis

#### Study design for microbiome data

We designed two randomized controlled longitudinal microbiome studies for this project: without butyrate treatment and with butyrate treatment. The study without butyrate treatment was designed to collect fecal samples of SOD1^G93A^ and wild-type mice over mouse ages of 1-, 2-, 3- and 4.25- month-old. It aimed to access the genetic effects on the dynamics of gut microbes. The study with butyrate treatment was designed to collect fecal samples of SOD1^G93A^ and wild-type mice over mouse ages of 1- (pre-treatment), 2- and 3- month-old. It aimed to access the butyrate treatment effects on the dynamics of gut microbes.

#### Fecal sample collection, sampling

We collected fecal samples for 16S rRNA sequencing. Fresh fecal samples from each group (SOD1^G93A^ and WT mice: for each time point, n = 5 from male and female mice) were collected from the colon and placed into the sterile tubes. The samples were kept with dry ice for low temperature and were sent to the University of Illinois at Chicago Research Resources Center for genomic sequencing. The genomic DNAs of samples were extracted using DNeasy Power Fecal Kit (12,830, Qiagen, Hilden, Germany) based on manufacturer’s instructions with a slight modification.

The samples were heated at 65°C for 10 min before homogenizing with FastPre-24 5 G bead-beating device (MP Biomedicals, Solon, OH, USA) at 6 m/s for 40 s. Genomic DNAs were fragmented into relatively small pieces (generally 250–600 bp fragments) before sequencing.

#### 16S rRNA sequencing

Genomic DNAs were PCR-amplified with the Earth Microbiome Project primers CS1_515 F and CS2_806 R targeting the V3–V4 regions of the 16S rRNA gene. A two-stage “targeted amplicon sequencing” protocol as described in^31^ was used to generate amplicons. Two independent PCR steps were conducted in the workflow for preparing samples for next-generation amplicon sequencing. In the first stage, PCR amplification was performed using primers containing CS1 and CS2 linkers (CS1_341 F: 50-ACACTGACGACATGGTTCTACAGTGCCAGCMGCCGCGGTAA-30; CS2_806 R: 50-TACGGTAGCAGAGACTTGGTCTGGACTACHVGGGTWTCTAAT-30) to the V3–V4 variable region of the 16S rRNA gene, while in the second stage, PCR amplification was performed on the first stage of PCR products using the Fluidigm Access Array barcoded primers. The 16S rRNA gene sequencing was performed using MiSeq according to the Illumina protocol. Based on the distribution of reads per barcode, the amplicons were pooled, re-pooled and purified. Then, the re-pooled libraries were loaded onto a Miniseq flow cell and sequenced (2x153 paired-end reads). Library preparation, pooling, and sequencing were performed at the Genome Research Core within the Research Resources Center at the University of Illinois Chicago.

#### Bioinformatic analysis

After DNA sequencing, all possible raw paired-end reads were evaluated and merged using the PEAR (Paired-End reAd mergeR) software (http://www.exelixis-lab.org/web/software/pear).^32^ To filter out low-quality reads, quality trimming was processed based on quality threshold (*p* = .01) and length parameters (minimum length = 225). The adapter/primer sequences were trimmed from the reads (fwd = GTGCCAGCMGCCGCGGTAA, and rev = GGACTACHVGGGTWTCTAAT) and ambiguous nucleotides (N = 1) were trimmed from the ends and reads with internal ambiguous nucleotides were discarded. Chimeric sequences are artifacts of the PCR process and occur when portions of two separate amplicons fuse during the amplification process. Sequencing chimera filtering was carried out using a standard chimera checking program (the reference-based USEARCH (http://www.drive5.com/usearch) algorithm)^32^ to identify and remove the chimeric sequences from the dataset as compared with a reference database (silva_132_16S.97).^8^ The sequences that passed both reference and algorithm methods are retained, and operational taxonomic units (OTUs) were selected based on a 97% similarity representing each OUT. Dereplication and sequences clustering via naïve read simplification (a.k.a. sub-OTU processing) techniques were used to reduce read count complexity. First, sequences were dereplicated; then minor sequences, those with counts below a given threshold (i.e., master cutoff of 10, and cluster threshold of 97% similarity) were clustered to major sequences. Then, OTUs were annotated using the USEARCH algorithm^33^ to compare with the reference database (silva _132_16S.97)^34^ to identify references and taxonomic assignment from kingdom to species.^35^

After removing the identical sequences with ≥97% identity of each other to make it nonredundant, the identified taxa have total counts of 4,369,887 (Min: 38.0, Max: 71,935.0, Median: 57,916.000 and Mean: 5,5315.025 per sample). On the phylum level, Bacteroidetes (total counts: 2,766,568, 63.31%) and Firmicutes (total counts: 1,407,939,32.22%) accounted for most of the microbial population in all samples (95.53%) and Proteobacteria accounted for 1.92% (total reads: 84,079), while all other taxa were less than 1%.

Before normalization, data are first filtered to remove read counts associated with particular taxa (i.e., Chloroplast, mitochondria, Mitochondria, Chloroplast), then read counts were normalized as fraction of total sequence counts in each sample to obtain relative sequence abundance. The OTUs were removed from downstream analysis if they were not present in all samples, had relative abundance less than 0.01%.

### Statistical analysis

All data are expressed as the mean ± SD. All statistical tests were two-sided. The *p* values < .05 were considered statistically significant. Before choosing an appropriate statistical test or model, the Shapiro-Wilk normality test for the distribution were performed for all the measurement variables. The differences between samples for two groups were analyzed using unpaired student’s t-test, Welch’s *t* test or Wilcoxon test based on the distribution of the testing variables. The differences between samples for more than two groups were analyzed using one-way ANOVA, two-way ANOVA or Kruskal-Wallis rank sum test as appropriate based on data distribution and the number of factors, respectively. The *p* values in ANOVA analyses and Kruskal-Wallis rank sum test were adjusted for correction of multiple comparisons using the Tukey or false discovery rate (FDR) method to ensure accurate results. Pairwise correlation analysis was conducted using Pearson or Spearman methods based on whether the testing variable was normally distributed or not. Regression plots in correlation analysis, line plots and scatter plots in longitudinal analysis were conducted using the ggplot2 package via R. To identify the core bacteria that were dynamically changed over time, we fitted two longitudinal models: Fast Zero-inflated Negative Binomial Mixed Modeling (FZINBMM)^36^ and the linear mixed-effects models.^37,38^ All the statistical analyses of microbiome data were performed using the R software (R version 4.0.4, 2021, The R Foundation for Statistical Computing Platform). Other statistical analyses were performed using GraphPad Prism 6 (GraphPad, Inc., San Diego, CA., USA).

## Results

### Slow intestinal mobility and weak muscle strengths correlated with aggregation of SOD1^G93A^ in intestine of the ALS mice

**Figure 1.**
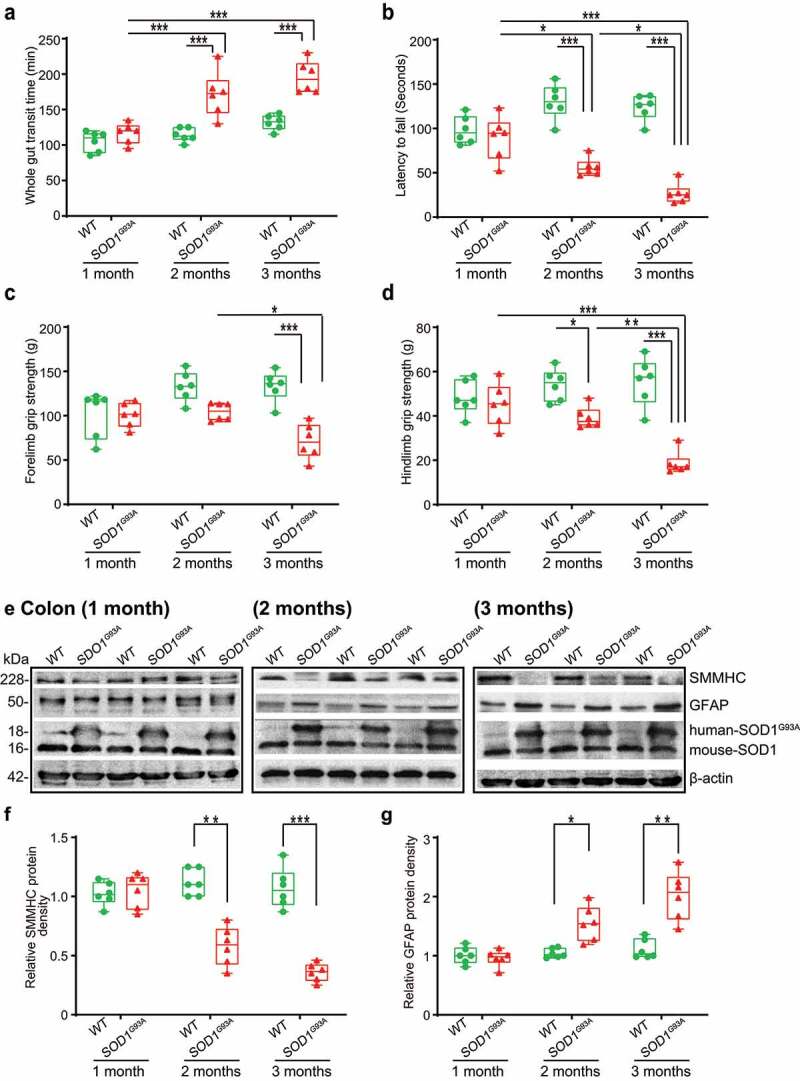
**SOD1^G93A^ mice have slow intestinal mobility, decreased rotarod test time and grip strength during ALS progression**. (a) SOD1^G93A^ mice significantly increased gut transit time starting at 2-month-old compared to WT mice. In age-matched WT and SOD1^G93A^ mice, intestinal mobility was tested using Evans blue marker (5% Evans blue, 5% gum Arabic in drinking water). (Data are expressed as mean ± SD. n = 6, two-way ANOVA test, ***P < .001, adjusted by the Tukey method). (b) Starting at 2-month-old, SOD1^G93A^ mice had significant reduced rotarod test time compared to WT mice. (Data are expressed as mean ± SD. n = 6, two-way ANOVA test, *P < .05, **P < .01, ***P < .001, adjusted by the Tukey method). (c) Forelimb grip strength in WT and SOD1^G93A^ mice at different time points and (d) Hindlimb grip strength in WT and SOD1^G93A^ mice at different time points. (Data are expressed as mean ± SD. n = 6, two-way ANOVA test, *P < .05, **P < .01, ***P < .001, adjusted by the Tukey method). (e) At the age of 2 months old, the expression of SMMHC protein started to decrease while the expression of GFAP protein started to increase in age matched SOD1^G93A^ mice compared to WT mice, and (f) and (g) Quantification for the expression of SMMHC and GFAP proteins in different time points. (Data are expressed as mean ± SD. n = 6, Kruskal-Wallis test with pairwise comparisons using Wilcoxon rank sum exact test, *P < .05, ***P < .01, ***P < .001 adjusted by the FDR method)

To study the intestinal changes before the onset of ALS symptoms, we assessed the intestinal transit time of SOD1^G93A^ mice at 1-, 2-, and 3-month-old, using a whole gut mobility assay.^39^ The 1-month-old SOD1^G93A^ mice showed no significant changes in mobility, compared with the wild-type (WT) mice. We found significantly increased gut transit time starting at 2-month-old (Figure 1a), suggesting slow intestinal mobility in the SOD1^G93A^ mice. The disturbance of mobility in ALS may reflect changes in smooth muscle function. We then examined the neuromuscular activity performance o*f* the SOD1^G93A^ mice on an accelerating rotarod.^40^ Muscle strength was evaluated using the grip strength and the hanging wire test. We tested their coordination and balance by their ability to ride rotarod. Starting at 2-month-old, SOD1^G93A^ mice had significantly reduced time on the rotarod, an indicator of weakened muscle function (Figure 1b). We further examined the forelimb grip strength of the SOD1^G93A^ mice at 1-, 2, and to 3- month-old and found time-dependently decreased forelimb grip strength (Figure 1c). Interestingly, decreased forelimb grip strength negatively correlated with increased time of intestinal mobility (r = −0.68, *p* = .015 in month 2 and r = −0.83, *p* < .0001 in month 3, respectively). We also found the similar reduction of hindlimb grip strengths overtime (Figure 1d). Decreased hindlimb grip strength was negatively correlated with increased time of intestinal mobility (r = −0.48, *p* = .1172 in month 2 and r = −0.83, *p* < .0001 in month 3, respectively).

### Altered enteric neuromuscular markers in the ALS G93A mice

At the protein level, we then examined the expression of the smooth muscle marker, smooth muscle myosin heavy chain (SMMHC),^41^ using western blots (WB) (Figure 1e). There was a significant reduction of SMMHC at the protein level in the 2-month-old SOD1^G93A^ mice (male and female) (Figures 1E & 1 F). There are enteric glia cells in the ENS and beneath the epithelium throughout the intestinal mucosa.^42^ A recent study showed that enteric glia cells regulate intestinal mobility.^43^ Therefore, we investigate the enteric glia by testing glial fibrillary acidic protein (GFAP) in SOD1^G93A^ mice at the ages of 1-, 2-, and 3-month-old (Figure 1e), whereas the endogenous mouse SOD1 remained the same. The GFAP expression was significantly enhanced in the 2-month-old and 3-month-old SOD1^G93A^ mice, whereas the endogenous mouse SOD1 remained the same (Figures 1F &1G). We also showed a significant increase of human SOD1^G93A^ in the intestine in the 2-month-old transgenic SOD1^G93A^ mice, and the changes of ENS markers were correlated with the increased human SOD1^G93A^ in the colon in the SOD1^G93A^ mice (Figure 1e).

### Altered enteric neuromuscular structure in the ALS G93A mice starting at 2-month-old

**Figure 2.**
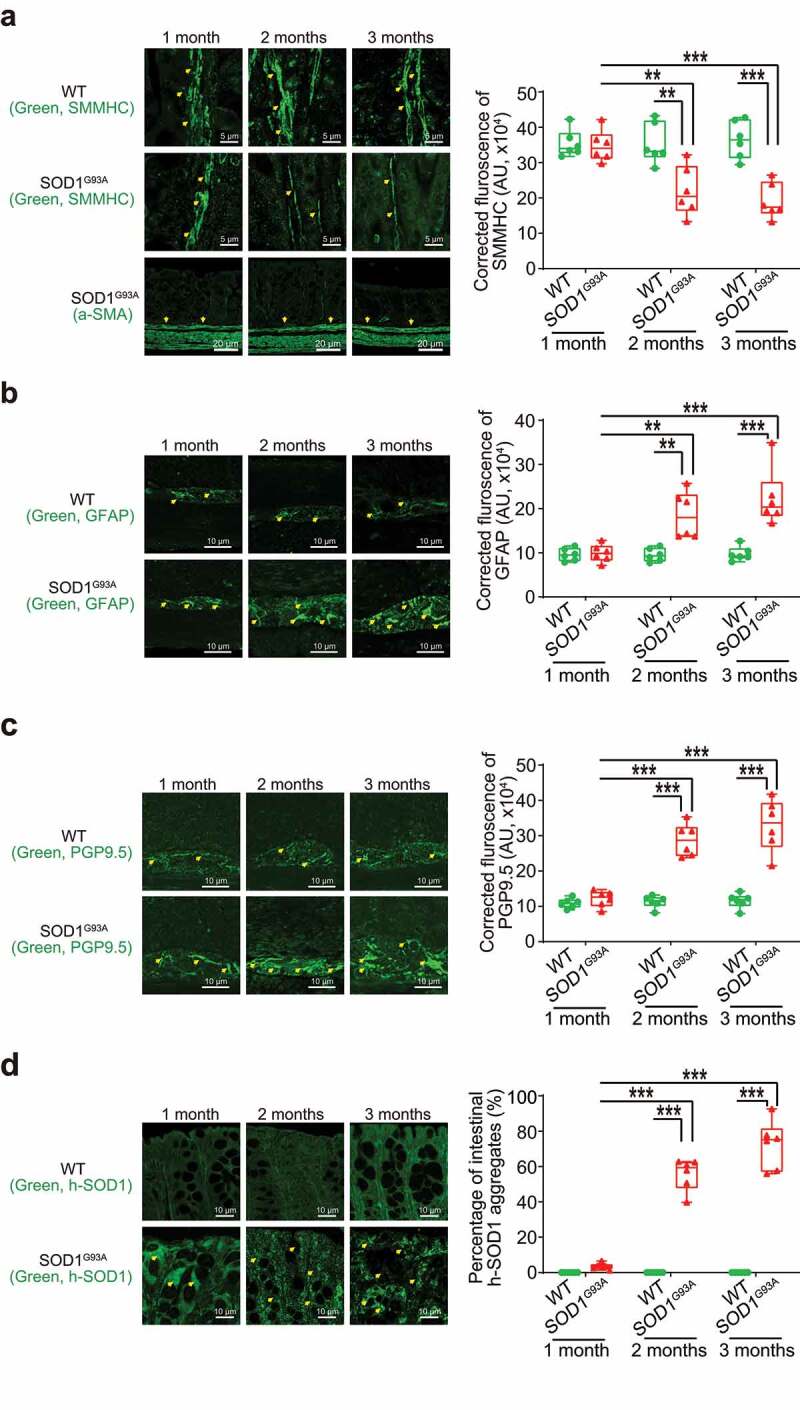
**Defects in the enteric nervous system structure in the SOD1^G93A^ mice starting at 2-month-old**. (a) SMMHC started decrease at 2-month-old in the colon of the SOD1^G93A^ mice compared to the aged-match WT mice in immunofluorescence staining and quantification of SMMHC staining. There is no change of alpha-smooth muscle actin in immunofluorescence staining. Images are from a single experiment representative of 6 mice per group. (Data are expressed as mean ± SD. n = 6, two-way ANOVA test, **P < .01, ***P < .001, adjusted by the Tukey method). (b) GFAP started to increase at 2-month-old in the colon of the SOD1^G93A^ mice compared to the age-matched WT mice in immunofluorescence staining and quantification of GFAP staining. Images are from a single experiment, representative of 6 mice per group. (Data are expressed as mean ± SD. n = 6, Kruskal-Wallis test, ***P < .01, ***P < .001, adjusted by the FDR method). (c) PGP9.5 was also enhanced in the SOD1^G93A^ mice at 2-month-old in the colon of the SOD1^G93A^ mice compared to the age-matched WT mice in immunofluorescence staining and quantification of PGP9.5 staining. Images are from a single experiment, representative of 6 mice per group. (Data are expressed as mean ± SD. n = 6, two-way ANOVA test, ***P < .001). (d) Aggregation of human-SOD1^G93A^ protein was observed starting from 2-month-old SOD1^G93A^ mice compared to the age-matched WT mice in immunofluorescence staining and human-SOD1 protein aggregates percentage analysis. Images are from a single experiment, representative of 6 mice per group. (Data are expressed as mean ± SD. n = 6, Kruskal-Wallis test with pairwise comparisons using Wilcoxon rank sum exact test, **P < .01, ***P < .001, adjusted by the FDR method)

Using immunostaining, we further detected the distribution of SMMHC (Smooth Muscle Myosin Heavy Chain) in the intestine of SOD1^G93A^ mice in different age groups (Figure 2a). Starting from 2-month-old, we were able to find the reduced SMMHC in the colon of the SOD1^G93A^ mice. In the meantime, whereas there is no change of α-SMA (alpha-Smooth Muscle Actin) in the colon of the SOD1^G93A^ mice (Figure 2a). In the 2-month-old SOD1^G93A^ mice, we found enhanced expression of GFAP (Glial Fibrillary Acidic Protein) (Figure 2b). The intracellular neuronal marker Protein Gene Product (PGP) 9.5 is a marker for neural-crest-derived precursor cells during gut development.^44^ G93A mice have increased PGP9.5 expression (Figure 2c). The distribution of human-G93A-SOD1 mutated protein was used as an indicator of ALS progression. Interestingly, we observed the aggregation of human-SOD1^G93A^ in the intestine, starting at the 2-month-old (Figure 2d).

### The aggregation of SOD1^G93A^ in the neurons and intestine of G93A mice

**Figure 3.**
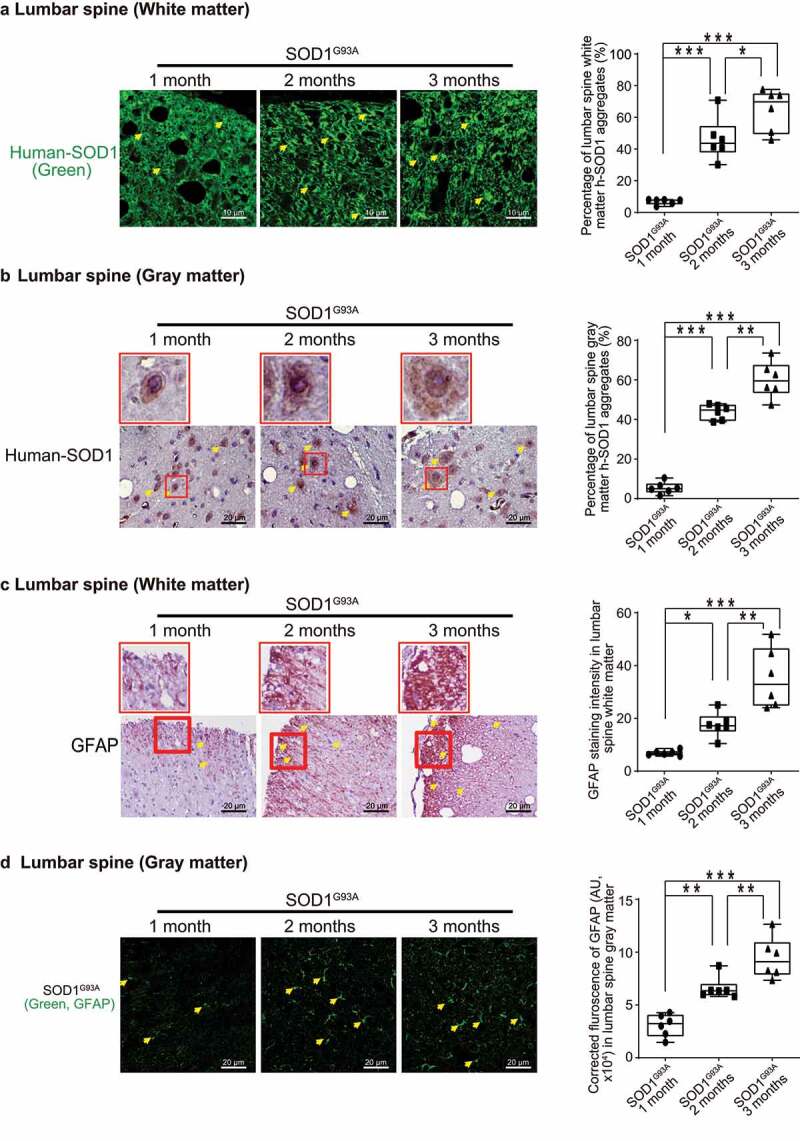
**Human SOD1^G93A^ protein was aggregated, and GFAP protein was increased during ALS progression**. Aggregation of human-SOD1^G93A^ protein was observed in the lumbar spine white matter (a) and gray matter (b) starting in 2-month-old SOD1^G93A^ mice in immunofluorescence staining, immunohistochemistry staining and human-SOD1 protein aggregates percentage analysis. Images are from a single experiment, representative of 6 mice per group. (Data are expressed as mean ± SD. n = 6, one-way ANOVA test, *P < .05, **P < .01, ***P < .001). GFAP expression was enhanced starting from 2-month-old in the lumbar spine white matter (c) and gray matter (d) in the SOD1^G93A^ mice in immunofluorescence staining, immunohistochemistry staining and GFAP staining quantification. Images are from a single experiment, representative of 6 mice per group. (Data are expressed as mean ± SD. n = 6, one-way ANOVA test, *P < .05, **P < .01, ***P < .001)

The aggregation of SOD1^G93A^ in the spinal cord and neurons is a molecular hallmark of ALS progression. The aggregation of human-SOD1^G93A^ protein was observed in the lumbar spine white matter and gray matter, starting in 2-month-old SOD1^G93A^ mice (Figure 3a & B). GFAP expression was also enhanced starting from 2-month-old in the white matter and gray matter of the lumbar spine in the SOD1^G93A^ mice (Figure 3c &D).

### Association of altered ENS and the increased aggregation of SOD1^G93A^ in SOD1^G93A^ mice in longitudinal studies

**Figure 4.**
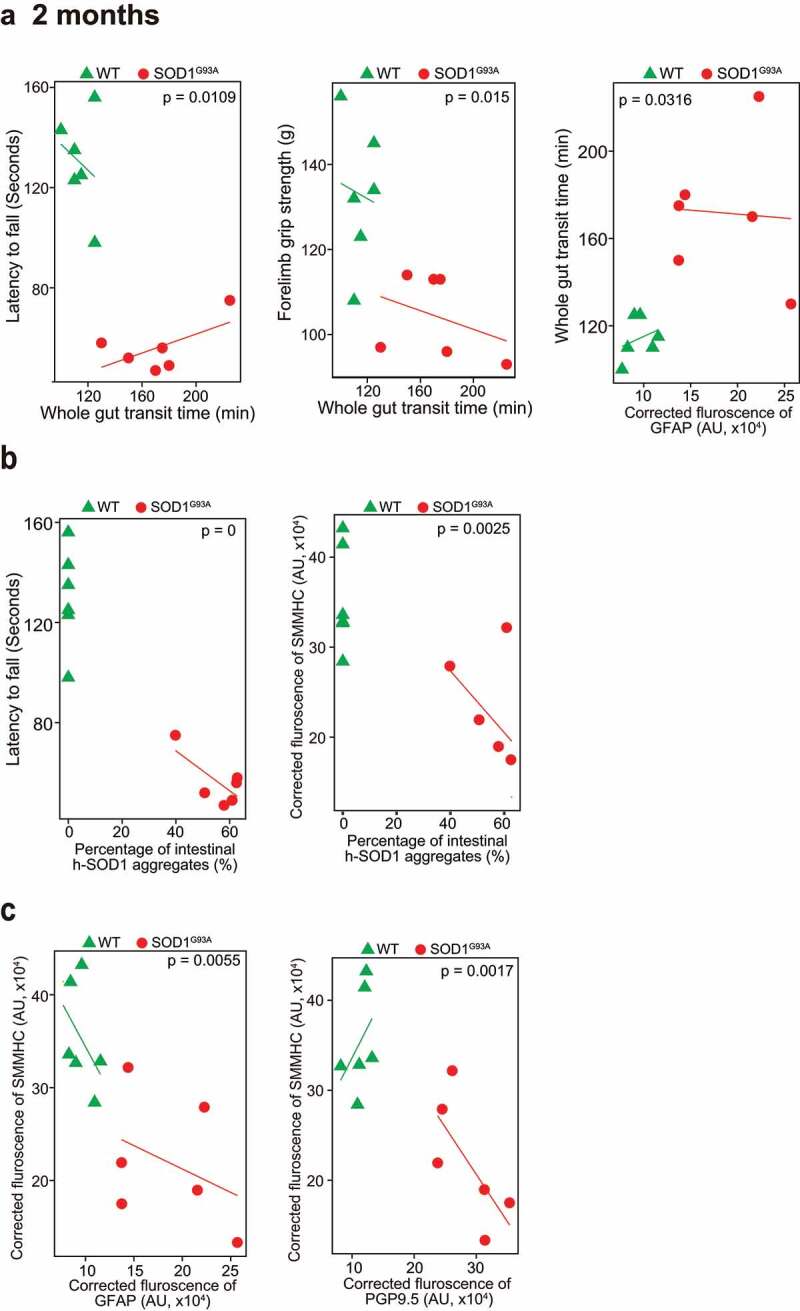
**Association of altered ENS and increased aggregation of human-SOD1^G93A^ in the SOD1^G93A^ mice in longitudinal studies**. (a) The correlation analysis between intestinal mobility (Whole gut transit time) and latency to fall, forelimb grip strength; staining intensity of GFAP, aggregation of human SOD1^G93A^ protein respectively in the 2-month-old SOD1^G93A^, compared with WT mice. (*P* values are labeled in figures, n = 6). Slow Intestinal mobility links with decreased forelimb grip strength, increased staining intensity of GFAP, increased aggregation of human SOD1^G93A^ protein respectively in 2-month-old SOD1^G93A^. (b) The correlation analysis between aggregation of human SOD1^G93A^ protein and latency to fall and staining intensity of SMMHC in 2-month-old SOD1^G93A^, compared with WT mice. (*P* values are labeled in figures, n = 6). Increased aggregation of human SOD1^G93A^ protein links with decreased muscle strength and decreased SMMHC in the 2-month-old SOD1^G93A^. (c) The correlation analysis between staining intensity of SMMHC and staining intensity of GFAP, staining intensity of PGP9.5 protein respectively in the 2-month-old SOD1^G93A^, compared with WT mice. (*P* values are labeled in figures, n = 6). Decreased staining intensity of SMMHC links with increased staining intensity of GFAP and PGP9.5 respectively in the 2-month-old SOD1^G93A^, compared with WT mice

Our longitudinal data at the functional, cellular, and protein levels showed the reduced intestinal mobility, weak muscle strengths, altered ENS markers, and enhanced aggregation of SOD1^G93A^ starting at the 2-month-old (Fig. S1). Furthermore, we did correlation data analysis and showed that slow intestinal mobility significantly correlated with weaker muscle strength, the changes of ENS marker GFAP, and an increased SOD1^G93A^ aggregation in the colon of the 2-month-old SOD1^G93A^ mice (Figure 4a). Immunohistochemistry staining and intestinal human-SOD1^G93A^ protein aggregates were further analyzed. We showed that a significant increase of human SOD1^G93A^ in the colon of the 2-month-old transgenic SOD1^G93A^ mice were correlated with the decreased muscle strength (e.g., latency to fall), and decreased SMMHC, respectively (Figure 4b). Decrease of SMMHC significantly linked with an increase of GFAP and PGP9.5 in the 2-month-old SOD1^G93A^ mice (Figure 4c). Moreover, the changes of SMMHC and GFAP, from 3-month-old ALS colon, were correlated with slower intestinal movement (weaker muscle) and increased SOD1^G93A^ aggregation in the G93A mice (Fig. S2).

### SOD1^G93A^ mice with butyrate treatment showed a significantly longer latency to fall in the rotarod test, reduced SOD1^G93A^ aggregation, and decreased GFAP expression

**Figure 5.**
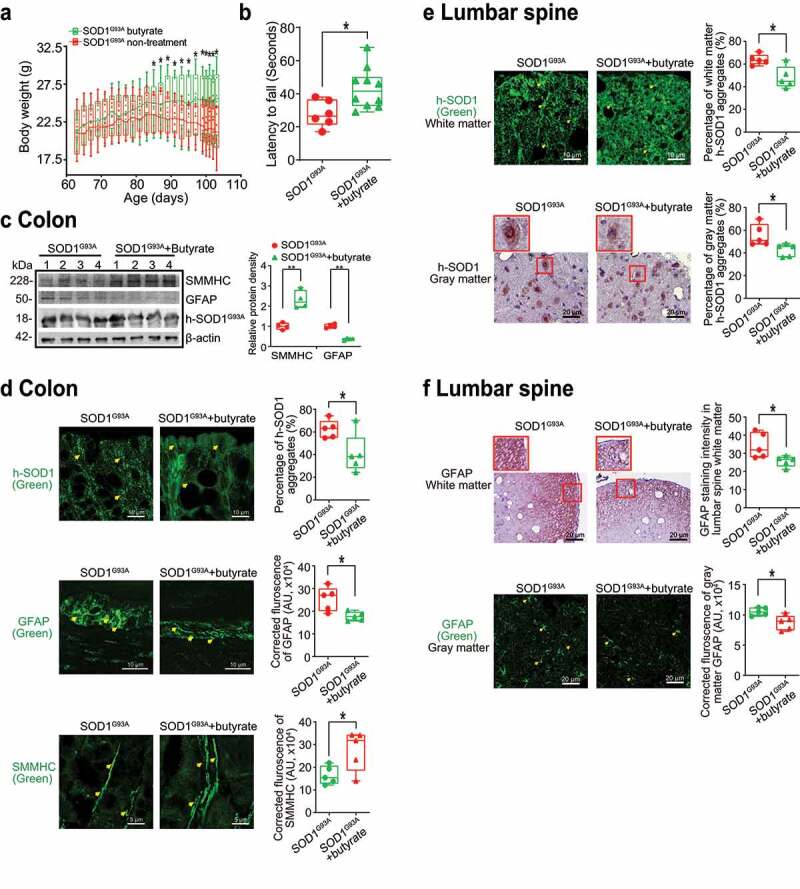
**Butyrate treatment led to reduced human-SOD1^G93A^ aggregation, and enhanced ENS and muscle function in intestine and neurons of SOD1^G93A^ mice**. (a) Body weight changes of SOD1^G93A^ mice after butyrate treatment. Male or female SOD1^G93A^ mice were treated with or without 2% butyrate in the drinking water starting from 63 days (2.1 months) to 101 days (3.4 months). Butyrate treated SOD1^G93A^ mice started to show less weight loss from the age of 83 days (1.4 months), compared to the no-treatment SOD1^G93A^ mice. Each data point represents the average body weight. (Data are expressed as mean ± SD. n = 6–10, two-way ANOVA test, *P < .05). (b) SOD1^G93A^ mice with butyrate treatment showed a significantly increased rotarod test time. At age 14–15 weeks, the mice were subjected to the trial on the accelerating spindle (4 to 40 rpm) for 300 seconds. Latency to fall was recorded when the mouse fell from the rod. Each mouse was tested in 4 trials per day for 2 consecutive days. The mean times for 8 trials of the tests were calculated for each mouse. (Data are expressed as mean ± SD. n = 6–10, Welch’s t-test, *P < .05). (c) Butyrate treatment led to increased SMMHC and decreased GFAP expression in SOD1^G93A^ mice. (Data are expressed as mean ± SD. n = 4, student t-test, **P < .01). (d) Reduced human-SOD1^G93A^ aggregation, decreased GFAP expression and enhanced SMMHC expression in intestine of SOD1^G93A^ mice with butyrate treatment. Images are from a single experiment and are representative of 5 mice per group. (Data are expressed as mean ± SD. n = 5, Welch’s t-test, *P < .05). (e) Reduced human-SOD1^G93A^ aggregation in lumbar spine of SOD1^G93A^ mice with butyrate treatment. Images are from a single experiment and are representative of 5 mice per group. (Data are expressed as mean ± SD. n = 5, Welch’s t-test, *P < .05).(f) Decreased GFAP expression in lumbar spine of SOD1^G93A^ mice with butyrate treatment. Images are from a single experiment and are representative of 5 mice per group. (Data are expressed as mean ± SD. n = 5, Welch’s *t*-test, *P < .05)

Our previous study has shown the beneficial role of butyrate in delaying the progress of ALS.^8^ Here, we investigated the role of butyrate treatment on muscle strength and ENS. Male and female SOD1^G93A^ mice were treated with or without 2% butyrate in the drinking water starting from 63 days (2.1 months). SOD1^G93A^ mice with butyrate treatment showed significantly higher body weight than the mice without treatment (Figure 5a). At age of 14- to 15- week-old (3.3–3.5 month-old), the mice were subjected to the trial on the accelerating spindle (4 to 40 rpm) for 300 seconds. When mice fell from the rod, we recorded their latency to fall. Mice were tested on the rod in 4 trials per day for 2 consecutive days. We found that SOD1^G93A^ mice with butyrate treatment had a significantly longer latency to fall in the rotarod test, compared to the SOD1^G93A^ mice without treatment (Figure 5b). Butyrate treatment led to increased SMMHC and decreased GFAP expression in SOD1^G93A^ mice (Figure 5c). We also found the reduced human-SOD1^G93A^ aggregation, decreased GFAP expression, and enhanced SMMHC expression in the intestines of SOD1^G93A^ mice with butyrate treatment (Figure 5d). In the lumbar spine of SOD1^G93A^ mice with a butyrate treatment, we found reduced human-SOD1^G93A^ aggregation (Figure 5e) and decreased GFAP expression (figure 5f). Taken together, these data suggested that butyrate treatment led to a significantly extended latency to fall in the rotarod test, reduced SOD1^G93A^ aggregation, and decreased GFAP expression.

### Antibiotics treatment led to reduced human-SOD1^G93A^ aggregation and enhanced ENS and muscle function in the intestine and neurons of SOD1^G93A^ mice

**Figure 6.**
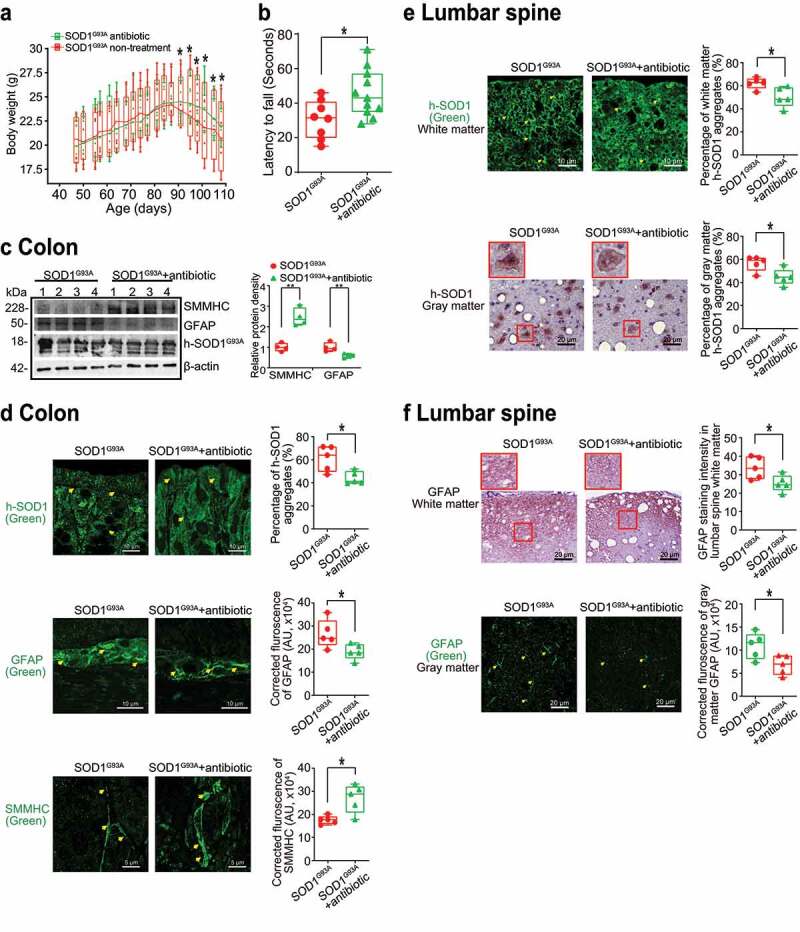
**Antibiotics treatment led to reduced human-SOD1^G93A^ aggregation, and enhanced ENS and muscle function in intestine and neurons of SOD1^G93A^ mice**. (a) Body weight changes of SOD1^G93A^ mice treated with antibiotics. Male or female SOD1^G93A^ mice were treated with or without antibiotics (1 mg/ml metronidazole and 0.3 mg/ml clindamycin) in the drinking water starting from 48 days (1.6 months) to 107 days (3.6 months). Antibiotic treated SOD1^G93A^ mice started to show less weight loss at the age of 91 days, compared to the non-treatment SOD1^G93A^ mice. Each data point represents the average body weight. (Data are expressed as mean ± SEM. n = 8–11, two-way ANOVA test, *P < .05). (b) SOD1^G93A^ mice with antibiotics treatment showed a significantly increased time in the rotarod test. At 15–16 weeks old, the mice were subjected to the trial on the accelerating spindle (4 to 40 rpm) for 300 seconds. Latency to fall was recorded when the mouse fell from the rod. Each mouse was tested in 4 trials per day for 2 consecutive days. The mean times for 8 trials of the tests were calculated for each mouse. (Data are expressed as mean ± SD. n = 8–11, Welch’s t-test, *P < .05). (c) Antibiotics treatment led to increased SMMHC and decreased GFAP expression in SOD1^G93A^ mice. (Data are expressed as mean ± SD. n = 4, student t-test, *P < .05, **P < .01). (d) Reduced human-SOD1^G93A^ aggregation, decreased GFAP expression and enhanced SMMHC expression in intestine of SOD1^G93A^ mice treatment with antibiotic. Images are from a single experiment and are representative of 5 mice per group. (Data are expressed as mean ± SD. n = 5, Welch’s t-test, *P < .05). (e) Reduced human-SOD1^G93A^ aggregation in lumbar spine of SOD1^G93A^ mice treatment with antibiotics. Images are from a single experiment and are representative of 5 mice per group. (Data are expressed as mean ± SD. n = 5, Welch’s t-test, *P < .05). (f) Decreased GFAP expression in lumbar spine of SOD1^G93A^ mice treatment with antibiotics. Images are from a single experiment and are representative of 5 mice per group. (Data are expressed as mean ± SD. n = 5, Welch’s *t*-test, *P < .05)

To test the role of manipulating microbiome in ALS, male and female SOD1^G93A^ mice were also treated with antibiotics^45^ (1 mg/ml metronidazole and 0.3 mg/ml clindamycin) in the drinking water starting from 48 day-old (1.6-month-old). The antibiotics treated mice had less weight loss, compared to mice without treatment (Figure 6a). At age 107 days (3.6 months), the mice were subjected to the trial on the accelerating spindle. Latency to fall was significantly longer with antibiotic treatment (Figure 6b). Furthermore, the antibiotic treatment led to increased SMMHC and decreased GFAP expression in SOD1^G93A^ mice, compared to those without treatment (Figure 6c). We also found reduced human-SOD1^G93A^ aggregation, decreased GFAP expression, and enhanced SMMHC expression in the intestines of SOD1^G93A^ mice treated with antibiotics (Figure 6d). We then tested human-SOD1^G93A^ aggregation and GFAP expression in the lumbar spine of SOD1^G93A^ mice treated with or without antibiotics. The antibiotic treatment restored the SMMHC (Figure 6e) and reduced GFAP (figure 6f). These data suggest that altered intestinal microbiome and function correlate with the skeletal muscle activity and motor neuron function in ALS.

### Increased aggregation of SOD1 mutant protein by fecal microbes in human colonoids

**Figure 7.**
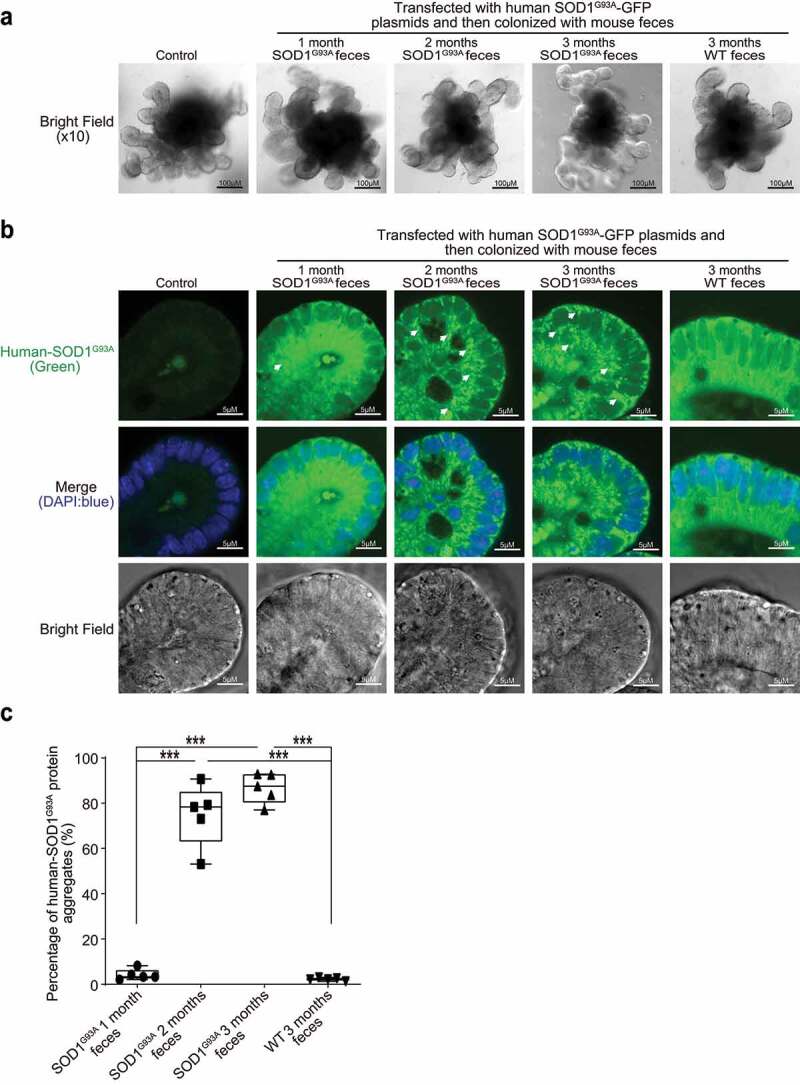
**Increased aggregation of SOD1 mutant protein in human colonoids transfected with SOD1^G93A^-GFP plasmids and then colonized with SOD1^G93A^ or WT mice feces**. (a) Human colonoids were transfected with SOD1^G93A^-GFP plasmids after 48 hours and treated with feces from SOD1^G93A/^WT mice at different ages for 2 hours. After 2-hour treatment of mice feces, the phenotype of human colonoids, e.g., shape and buds did not change. Images are representative of experiments in triplicate. (b) Human SOD1 expression in human colonoids were transfected with SOD1^G93A^-GFP plasmids and then colonized with feces from SOD1^G93A^ or WT mice. Images are representative of experiments in triplicate. (c) More SOD1^G93A^ protein aggregations in the human colonoids after SOD1^G93A^-GFP plasmid transfection and 2 and 3-month-old SOD1^G93A^ mice feces colonization. (Data are expressed as mean ± SD. n = 5, one-way ANOVA test, ***P < .001)

Colonoids derived from intestinal stem cells can assess host-microbial interactions, as we performed in previous studies.^46–49^ First, we transfected human colonoids with SOD1^G93A^-GFP plasmids after 48 hours, then treated with feces from WT or SOD1^G93A^ mice at different ages for 2 hours (Figure 7). Over time the colonoids were imaged (Figure 7a), then collected for immunostaining and western blots. We did not see significant changes in the number of buds or size of organoids after treatment, as shown in Figure 7a. Interestingly, we found that feces from the ALS mice could induce SOD1 aggregation in human colonoids transfected with SOD1^G93A^-GFP plasmids (Figure 7b). The feces of 2- and 3-month-old SOD1^G93A^ mice generated more SOD1^G93A^ protein aggregates (Figure 7c), suggesting the direct effect of microbes in the feces of SOD1^G93A^ mice in increasing SOD1^G93A^ protein aggregation. It also indicated the age-dependent functional differences of the microbiome in the SOD1^G93A^ mice.

### Changes of fecal microbiome in SOD1^G93A^ mice

To identify the changes of bacterial community in the feces of the SOD1^G93A^ mice, we performed the longitudinal data analysis of the microbiome data from 1-, 2-, and 3- month-old mice, compared with the age-matched WT mice. At the species level, we found the significantly different changes of certain bacteria between groups in a time-dependent manner (Table 1). We used different models (e.g., Fast Zero-inflated Negative Binomial Mixed Modeling^50^ and linear mixed-effects models^37,38^) to examine the changes of microbiome at the species level, as indicated in Table 1.

**Table 1.** Bacteria species identified by the longitudinal study of SOD1^G93A^ mice and age-matched WT mice [the adjusted *p*-values (adj-p) associated comparing group, time, and group and time interaction]

Species	Group overall (adj-p)	2-month (adj-p)#	3-month (adj-p)#	4.25-month (adj-p)#	Group at 2-month (adj-p)&	Group at 3-month (adj-p)&	Group at 4.25-month (adj-p)&	Model*
Enterorhabdus muris	0.0217	0.9664	0.4353	0.1337	0.1917	0.1917	0.1917	Model 1
Enterorhabdus muris	0.0797	0.9176	0.5008	0.1577	0.6586	4e-04	0.6291	Model 2
Bacteroides thetaiotaomicron	0	0.8829	0.0973	0.0655	0	0	0	Model 1
Bacteroides thetaiotaomicron	0.5014	0.9153	0.8655	0.011	0.9336	0.1032	0.6291	Model 2
Parabacteroides goldsteinii CL02T12 C30	0.1759	0.6241	0.2902	0.0041	0.5201	0.5201	0.5201	Model 1
Parabacteroides goldsteinii CL02T12C30	0.5014	0.9587	0.5008	0.011	0.6586	0.2131	0.9353	Model 2
Staphylococcus sp. UAsDu23	0.5206	0.0067	0.8223	0.8483	0.0448	0.0448	0.0448	Model 1
Lactobacillus gasseri	0.0217	0.9218	0.1073	0.1318	0.0027	0.0027	0.0027	Model 1
Lachnospiraceae bacterium A4	0.0936	0.5104	0.4353	0.0049	0.876	0.876	0.876	Model 1
Lachnospiraceae bacterium A4	0.0797	0.3504	0.3825	0.011	0.6586	0.2131	0.1776	Model 2
Clostridium sp. ASF502	0.0201	0.0055	7e-04	0.0041	0.1108	0.1108	0.1108	Model 1
Clostridium sp. ASF502	0.0955	0.1215	0.0218	0.0403	0.486	0.0468	0.1776	Model 2
Anaerotruncus sp. G3(2012)	0.0217	0.0011	3e-04	0.0049	0.1108	0.1108	0.1108	Model 1
Anaerotruncus sp. G3(2012)	0.423	0.1215	0.0375	0.2637	0.6586	0.2131	0.6291	Model 2
Clostridium sp. Culture Jar-8	0.4552	0.4874	0.3825	0.011	0.6183	0.9971	0.1851	Model 2
Bacteroides acidifaciens	0.423	0.9587	0.5008	0.0403	0.6586	0.0573	0.9353	Model 2
Desulfovibrio fairfieldensis	0.2087	0.9176	0.465	0.6766	0.6586	0.0468	0.9353	Model 2

*Model 1 was fitted by Fast Zero-inflated Negative Binomial Mixed Modeling (FZINBMM) using group, time and their interaction term as fixed effects and intercept as random effect. The within-subject correlation of AR(1) was used to account for within-individual correlation and the log(total read counts in sample) was used as the offset to adjust for library sizes.

Model 2 was fitted by the linear mixed-effects models using group, time and their interaction term as fixed effects and intercept as random effect. The within-subject correlation of AR(1) was used to account for within-individual correlation and the arcsine square root transformation of taxa abundance counts was used in lieu of normalization.

# The adjusted *p*-values at 2-, 3-, and 4.25- months were obtained by comparing baseline 1-month.

& The adjusted *p*-values for groups at 2-, 3-, and 4.25- months were obtained by comparing group at 1-month.

**Figure 8.**
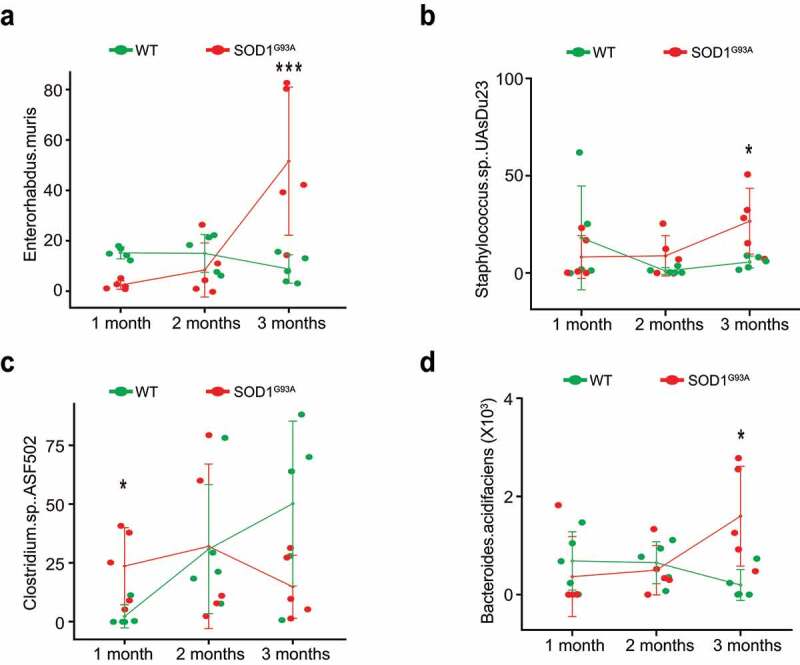
**Fecal microbiome changes in SOD1^G93A^ mice**. (a) Enterohabdus Muris started increasing in 2-month-old SOD^G93A^, and then significantly enhanced in 3-month-old SOD1^G93A^ mice, compared with WT mice. (n = 5, two-way Kruskal-Wallis rank sum test, ***P < .05 adjusted by FDR.). (b) Staphylococcus. Sp. UAsDu23 started increase in 2-month-old SOD^G93A,^ compared with 1-month-old SOD^G93A^ mice, and then significantly enhanced in 3-month-old SOD1^G93A^ mice, compared with WT mice. (n = 5, Welch’s t-test, *P < .05). (c) Clostridium sp. ASF502 enhanced in 1 months SOD^G93A^, compared with WT mice (n = 5, Welch’s t-test, *P < .05). (d) *Bacteroides acidifaciens* significantly enhanced in 3-month-old SOD1^G93A^ mice, compared with the WT mice. (n = 5, two-way ANOVA test, *P < .05, adjusted by the Tukey method)

To confirm the identified significant changes of bacteria by the longitudinal models and visualize the changes over time, we performed the line plots (Figure 8) and conducted ANOVA, Kruskal-Wallis rank sum test, Welch’s t-test or Wilcoxon test as appropriate for specific bacteria. Specifically, immune-related *Enterohabdus Muris* started increasing in the 2-month-old SOD1^G93A^, compared with the 1-month-old SOD1^G93A^ mice, and further significantly enhanced in the 3-month-old SOD1^G93A^ mice (Figure 8a). *Staphylococcus. Sp. UAsDu23* was enhanced in 1-month-old SOD1^G93A^ mice, and then significantly enhanced over time, compared with the age matched WT mice (Figure 8b). *Clostridium sp. ASF502* was significantly reduced in the 3-month-old SOD1^G93A^, compared with WT mice (Figure 8c). *Bacteroides acidifaciens* started to increase in the 2-month-old SOD1^G93A,^ compared with 1-month-old SOD1^G93A^ mice and was significantly enhanced in the 3-month-old SOD1^G93A^ mice, compared with WT mice. In contrast, the relative abundance of *Bacteroides acidifaciens* was stable in the WT mice over 2 months and reduced in the 3-month-old WT mice (Figure 8d). The dramatic changes of bacteria in the SOD1^G93A^ mice may explain the ability of the feces to enhance SOD1 aggregation in the colonoids.

### Butyrate treatment reduced the microbial differentiation between WT and G93A mice

Interestingly, butyrate treatment reduced the changes in the fecal microbiome in the SOD1^G93A^ mice (Table 2). To identify the altered bacteria, we used the appropriate statistical methods, based on study design (e.g., one-way or two-way factors, two-group or more than two-group comparisons) and data distributions (e.g., normal or non-normal via the Shapiro-Wilk normality test). For example, the significantly enhanced *Enterohabdus Muris* in 3-month-old SOD1G93A mice was not significant anymore after butyrate treatment, compared with WT mice (n = 6, two-way Kruskal-Wallis rank sum test, ***P < .2 adjusted by false discovery rate.) The significantly enhanced *Staphylococcus. Sp. UAsDu23* in 12-week-old SOD1^G93A^ mice without butyrate treatment was not significant after butyrate treatment (n = 6, Welch’s t-test, *P* = .55). The identified Clostridium sp. ASF502 in 1-month-old SOD^G93A^, compared with 1-month-old WT mice, was no longer significant after butyrate treatment (n = 6, Wilcoxon rank sum test, *P* = .6). The significantly enhanced *Desulfovibrio fairfieldensis* in the 3-month-old SOD1^G93A^ mice was no longer significant, compared with the WT mice (n = 6, Kruskal-Wallis rank sum test, *P* = .4 adjusted by false discovery rate). Instead, butyrate treatment enhanced *Lachnospiraceae bacterium* A4 but remained the same without treatment in the 3-month-old SOD1^G93A^ mice (n = 6, Kruskal-Wallis rank sum test, *P* = .03). Without butyrate, at 1-month-old, *Candidatus Arthromitus sp. SFB.mouse.NYU*, a bacterium in regulating Th17 immunity,^51^ in the SOD1^G93A^ mice, was significantly different from the WT (ANOVA with Tukey adjusted *p* = .048), but there is no longer a significant difference with butyrate treatment. These data indicate the beneficial role of butyrate in restoring the microbiome and delaying the ALS progression in SOD1^G93A^ mice.

**Table 2.** Species identified by the longitudinal study in SOD1^G93A^ mice and age-matched WT mice with butyrate treatment [the adjusted *p*-values (adj-p) associated comparing group, time, and group and time interaction]

Species	Group overall (adj-p)	2-month (adj-p)#	3-month (adj-p)#	Group at 2-month (adj-p)&	Group at 3-month (adj-p)&	Model*
D_6__Parabacteroides goldsteinii CL02T12C30	0.777	0.002	0.6093	0.1302	0.867	Model 1
D_6__Parabacteroides goldsteinii CL02T12C30	0.9366	0.0106	0.928	0.1848	0.9547	Model 2
D_6__Clostridium sp. ASF356	0.6512	0.287	0.0164	0.7647	0.1022	Model 1
D_6__Clostridium sp. ASF356	0.5862	0.2137	0.024	0.7731	0.1868	Model 2
D_6__Clostridium sp. ASF502	0.7548	0.003	0.0011	0.7647	0.867	Model 1
D_6__Clostridium sp. ASF502	0.9366	0.0713	0.0204	0.8169	0.7351	Model 2
D_6__Clostridium sp. Culture Jar-8	0.0826	0.219	0.8062	0.056	0.3042	Model 1
D_6__bacterium enrichment culture clone M244	0.9366	0.3992	0.0443	0.9329	0.7351	Model 2

*Model 1 was fitted by Fast Zero-inflated Negative Binomial Mixed Modeling (FZINBMM) using group, time and their interaction term as fixed effects and intercept as random effect. The within-subject correlation of AR(1) was used to account for within-individual correlation and the log(total read counts in sample) was used as the offset to adjust for library sizes.

Model 2 was fitted by the linear mixed-effects models using group, time and their interaction term as fixed effects and intercept as random effect. The within-subject correlation of AR(1) was used to account for within-individual correlation and the arcsine square root transformation of taxa abundance counts was used in lieu of normalization.

# The adjusted *p*-values at 2- and 3- months were obtained by comparing pretreatment 1 month.

& The adjusted *p*-values for groups at 2- and 3- months were obtained by comparing group at pretreatment 1-month.

## Discussion

**Figure 9.**
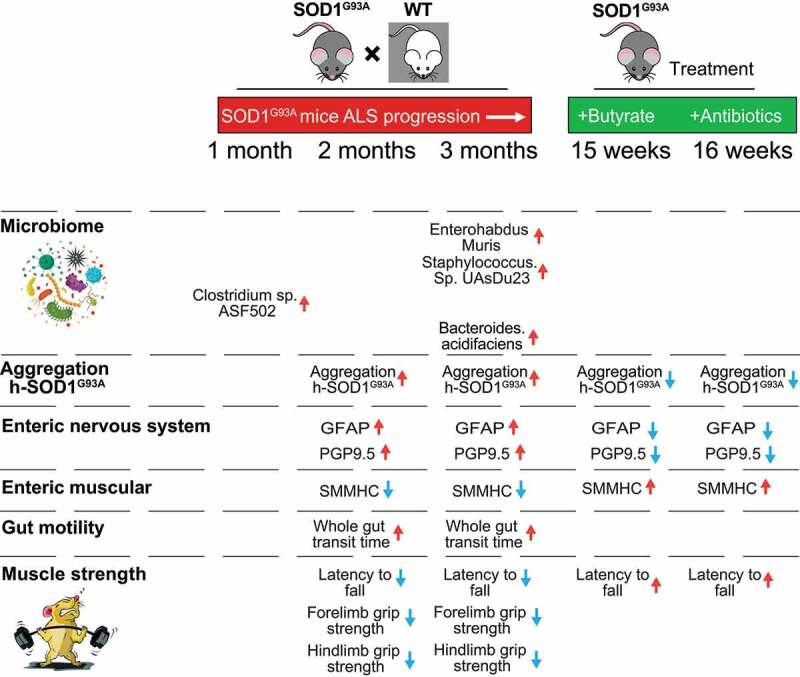
Timeline of altered microbiome, SOD1^G93A^ aggregation, EN function, and lumbar spine pathophysiological in the progression of SOD1^G93A^ ALS mice

In the current study, we have demonstrated a novel link between intestinal mobility, ENS, and microbiome in the SOD1 aggregation and progression of ALS. Dysbiosis occurred at the early stage of the SOD1^G93A^ mice (1-month-old) before observed dysfunction of ENS. The timeline for the microbiome changes, ENS, SOD1 aggregation in different organs, and muscle strength indicate the microbial difference in the 1-month-old ALS mice, compared to the WT mice (Figure 9). The SOD1^G93A^ mice aged one-month-old showed no significant changes of ENS and mobility but altered gut microbiome (e.g. increased *Clostridium sp. ASF502*), compared with the WT mice. At 2-month-old before ALS onset, SOD1^G93A^ mice had significant lower intestinal mobility, decreased grip strength, and reduced time in the rotarod. These changes correlated with consistent dysbiosis and increased aggregation of mutated SOD1^G93A^ in the colon and small intestine. We observed increased GFAP and decreased SMMHC expression. At the ALS onset period (3-month-old), G93A mice had much slower intestinal mobility, decreased grip strength, and reduced time in the rotarod test. SOD1^G93A^ mice had increased GFAP and decrease SMMHC expression. Human-G93A-SOD1 mutated protein showed severe aggregation in the intestine. Moreover, we found that butyrate treatment and antibiotic treatment restored intestinal mucosal function and corrected dysbiosis in the ALS mice. Manipulating the microbiome improves the muscle performance of SOD1^G93A^ mice. Our study provides insights into the fundamentals of intestinal neuromuscular structure/function and the microbiome in ALS.

Our longitudinal studies in the ALS human-SOD1^G93A^ mice have revealed microbial communities closely tied to host metabolism and immunity in ALS. We have previously reported the significant reduction of butyrate-producing bacteria, e.g., Roseburia (family *Lachnospiraceae*, order *Clostridiales*), in the SOD1^G93A^ mice.^8,9^ Butyrate-producing bacteria are known to play an important role in controlling gut inflammatory processes and the maturation of the immune system, primarily through the production of butyrate.^52^ Here, we further identified bacteria that are important for the immunity and inflammatory response at the species level. For example, an IBD model successfully isolated *Enterohabdus Murisi.*^53^ Reports indicate that *Lachnospiraceae bacterium A4* bacteria inhibits Th2-cell differentiation by inducing dendritic cell production of TGF-β.^54^ *Clostridium sp. ASF502* belongs to altered Schaedler flora^55^(ASF), a model microbial community with relevance *in vivo* and *in vitro*. It plays an essential role in immune system development, opportunistic pathogen resistance, GI function, and health in ASF mice. *Candidatus Arthromitus* sp. SFB-mouse-NYU directly accumulates pro-inflammatory Th17 cells into the intestinal lamina propria.^51^ *Candidatus Arthromitus.sp. SFB.mouse.NYU* in the 1-month-old G93A mice without butyrate is significantly different from the WT mice, but there was no significant difference after butyrate treatment. Butyrate-treated SOD1^G93A^ mice have reduced *Desulfovibrio fairfieldensis. Desulfovibrio fairfieldensis* is among the sulfate-reducing bacteria, which are anaerobic microorganisms that conduct dissimilatory sulfate reduction to obtain energy, resulting in the release of a significant quantity of sulfide. Altered sulfate-reducing bacteria are reported in human IBD cases.^56^ Our data suggested that the early ALS-associated dysbiosis of the intestinal microbiome is related to host metabolism, immunity, and microbial manipulation. Treatment with butyrate or antibiotics could slow ALS disease progression.

Here, we define the changes in intestinal microbiome and function during ALS progression, and their correlation with the progressive decline of skeletal muscle activity and motor neuron. A previous study has shown correlations between bacterial species abundances and transit times are diet dependent.^18^ This study used a gnotobiotic mouse model that mimics short-term dietary changes when humans travel to places with different culinary traditions in order to understand how different diets, microbiota, and the ENS interact to regulate gut mobility. The levels of unconjugated bile acids-generated by bacterial bile salt hydrolases-correlated with faster transit, including during consumption of a Bangladeshi diet. This study demonstrated how a single food ingredient interacts with a functional microbiota trait to regulate intestinal mobility.^18^ Here, we showed that butyrate, a bacterial product, significantly restored the ENS and smooth muscle in the ALS mice. Manipulating the microbiome by antibiotics also facilitates the ENS and muscle, thus delaying ALS onset and progression. An ALS study also indicates the temporal evolution of the microbiome, immune system, and epigenome with the disease progression of the SOD1^G93A^ mice.^15^ Our data in the ALS model and human colonoids have provided novel links among microbiome, SOD1 aggregation, and intestinal dysfunction. Dysbiosis and SOD1 mutated aggregation occurred at the early stage of the SOD1^G93A^ mice before observed slow intestinal mobility and dysfunction of ENS, suggesting a biomarker for ALS diagnosis. Our data further suggest that restoring a healthy microbiome reduced aggregation of the SOD1 G93A-mutated protein in the intestine and nervous tissues and slowed down the progression of ALS in mice.

On the one hand, it is unknown whether intestinal epithelial damage directly affects the enteric neurons and smooth muscles. On the other hand, the ENS is an important regulator of the proliferation and differentiation of the mucosal epithelium.^57^ Interestingly, the transgenic mice with human ALS mutation Prp-TDP43 A315T^58^ had a defect in enteric neurons, which may be associated with the microbiome and mucosal barrier abnormalities. In the future study, we will investigate the role of other ALS risk genes on the microbiome and ENS. The mechanism of mobility dysfunction depends on the intestinal region and the stage of diseases.^59,60^ There is an age-dependent shift in macrophage polarization that causes inflammation-mediated degeneration of the ENS.^61^ Further studies on pro-inflammatory serum cytokines and LPS combined with microbiome changes in patients will help with the early diagnosis of the disease.

To reconstruct the intestinal microflora in ALS patients, fecal microbiota transplantation (FMT) has been used to transfer the gut microbiota from healthy individuals to patients,^62^ as proved as an effective treatment for recurrent *Clostridium difficile* infection.^63^ Studies have showed that FMT could improve gastrointestinal and behavioral symptoms in neurological diseases, e.g. autism and Parkinson’s disease. The autism patients were treated with FMT daily for 7–8 weeks by mixing standardized human gut microbiota with a drink or via enema, and the improvement persisted 2 years after treatment^64,65^. Parkinson’s disease patients after one-week treatment of FMT improved constipation and motor symptoms such as leg tremors. The tremors recurred 2 months after FMT, whereas constipation was relieved even after 3 months.^66^ In ALS, gut microbiota dysbiosis facilitate the disease onset or drive its progression and related outcomes, in the presence of other risk factors. Intestinal bacterial flora as an external trigger could explain the rare cases of ALS in spouses or in some clusters.^67^ To treat gut microbiota dysbiosis through microbiota restoration would have the potential to interfere and slow ALS progression.^68^ Currently, ClinicalTrials.gov (https://clinicaltrials.gov/ct2/show/NCT03766321) has an ongoing ALS patients FMT trial for a year with 42 ALS patients, at an early stage 28 FMT-treated patients vs. 14 controls. This will promote a further understanding of microbiota restoration function.

Sporadic (SALS) and familial (FALS) forms of ALS manifest similar pathological and clinical phenotypes, suggesting that different initiating causes lead to a mechanistically similar neurodegenerative pathway. Some clinical studies have indicated intestinal abnormalities in ALS patients.^69–71^ ALS patients have delayed colonic transit time and gastric emptying, and up to half of them have constipation during the disease course.^71–73^ The changes in the intermediolateral columns and the Onuf nucleus in ALS have been detected. Increased LPS is reported in SALS patients.^5^ ALS patients have elevated intestinal inflammation and dysbiosis.^10,11^ The ENS and smooth muscle automatism are unable to modulate the motor functions of the digestive tract, which provide an anatomical explanation for these clinical manifestations. The therapeutic methods to target microbiome and intestinal functions, e.g, FMT, prebiotics, and probiotics, will help both SALS and FALS patients.

Currently, treatment with the FDA-approved drug, Riluzole merely extends the patient’s life span for a few months. The second new drug Radicava gained approval in 2017. However, there is still a need to develop new treatments for alleviating disease progression and improving the life quality of ALS patients. A phase 2 randomized, placebo-controlled trial treated patients for 24 weeks, which involved 137 ALS patients, 89 of whom received treatment with the combination of sodium phenylbutyrate and taurursodiol.^74^ It found a modest reduction in functional decline in patients receiving the combination therapy,^74^ suggesting the promising role of sodium phenylbutyrate. However, this study has no data on the changes in the human microbiome. A recent study reported the microbiota changes as a potential human ALS biomarker and suggested that targeting the microbiome could be considered to restore the status of the intestine.^14^ A larger trial testing more patients over a more extended period is also needed.

The limit of the current study is that there is no ENS data in human ALS. The direct interactions between ENS and the microbiome are unknown. Although human ALS and ENS functions have yet to be investigated, the insights gained from the analysis of the ALS mice and microbiome will contribute to a better understanding of how the microbiome affects the host in the progression of a disease. The direct role of other microbial metabolites, in addition to butyrate, on the function of the ENS and its mechanisms are also in the future plan.

## Conclusion

We have demonstrated a novel link between the microbiome, SOD1 aggregation, and intestinal mobility. Dysbiosis at the early stage of ALS progression in G93A mice occurs before observed dysfunction of the enteric neuromuscular system. SOD1 mutated aggregation occurred in both the intestine and spinal cord. Interestingly, fecal samples from the G93A mice were able to induce SOD1 mutated aggregation in human colonoids. Targeting the intestinal microbiome was able to improve neuromuscular function in ALS. Our study provides insights into the fundamentals of intestinal neuromuscular structure/function and the microbiome in ALS, suggesting the potential to use microbial biomarkers for the diagnosis and to manipulate the intestinal microbiome for the treatment.

## Data sharing statement

The authors confirm that the data supporting the findings of this study are available within the article and its supplementary materials. The data that support the findings of this study are also available from the corresponding author, JS, upon reasonable request.
